# The effect of cult-active medium on pregnancy outcomes after intracytoplasmic sperm injection in azoospermic men: A case-control study

**DOI:** 10.18502/ijrm.v19i12.10056

**Published:** 2022-01-12

**Authors:** Elham Asa, Rahil Janatifar, Seyedeh Saeideh Sahraei, Atefeh Verdi, Naser Kalhor

**Affiliations:** ^1^Department of Reproductive Biology, Academic Center for Education, Culture, and Research (ACECR), Qom, Iran.; ^2^Department of Mesenchymal Stem Cells, Academic Center for Education, Culture, and Research (ACECR), Qom, Iran.

**Keywords:** Calcium ionophore, Obstructive azoospermia, Fertilization, ICSI.

## Abstract

**Background:**

Failed oocyte activation following intracytoplasmic sperm injection (ICSI) as a result of calcium deficiency is a major challenge.

**Objective:**

We compared the effect of cult-active medium (CAM) on ICSI outcomes in obstructive azoospermia cases.

**Materials and Methods:**

The present study was conducted with 152 ICSI cases, classified into CAM and control groups. The injected oocytes in the control group were cultured in the cleavage medium, while in the artificial oocyte activation group, oocytes were chemically activated through exposure to 200 µL of CAM for 15 min. Fertilization and cleavage rates, quality of embryos, and biochemical pregnancy and live birth rates were assessed in both groups.

**Results:**

There were significant differences between the groups in terms of fertilization and cleavage rates after using the CAM in the percutaneous epididymal sperm aspiration (PESA) subgroup (p = 0.05, p ≤ 0.001) and in the testicular sperm extraction subgroup (p = 0.02, p = 0.04), compared to their control groups. Also, the pregnancy rate was significantly higher in the PESA-CAM subgroup (p = 0.03). The PESA-CAM subgroup demonstrated a significant difference in embryo quality after ICSI (p = 0.04). Unsuccessful embryo transfer and abortion were lower in both subgroups compared to the control groups, but this difference was not significant. Surprisingly, live birth rate was higher in the PESA-CAM subgroup (p = 0.03).

**Conclusion:**

CAM treatment could improve fertilization and cleavage rates in obstructive azoospermia participants. It had a significant effect on embryo quality, and pregnancy and live birth rates in PESA cases.

## 1. Introduction

Azoospermia (a lack of sperm in the semen) is a severe form of male infertility and it accounts for almost 10-15% of all male infertility. This condition can be divided into two groups: obstructive azoospermia (OA) and non-OA. In OA, spermatogenesis is normal, but there is occlusion or partial absence of the reproductive tract, while in non-OA, spermatogenesis is defective (1). Intracytoplasmic sperm injection (ICSI) has been widely used to treat severe male infertility, such as OA (2). However, fertilization failure occurs in 1-5% of ICSI cycles that may be explained by defects in the oocyte, sperm, or ICSI procedure. The source of intracellular calcium oscillations is endoplasmic reticulum that releases into the ooplasm after sperm entry (3). The calcium oscillations involved in the process of fertilization undergo a gradual decline in amplitude and frequency in a species-specific manner until the end of pronucleus formation. Since calcium is a universal secondary messenger in cells, controlling diverse biological processes, including proliferation, differentiation, axis formation, transcriptional activation, and apoptosis (4, 5), it plays a major role in fertilization. Sperm have a specific phospholipase C (PLC) isoform, called PLC-zeta, which is present at a specific concentration to increase the calcium level in the oocyte through the inositol-1, 4, 5-trisphosphate-mediated pathway. Therefore, sperm trigger an increase in the concentration of intracellular calcium (Ca ${}^{2+}$ ), activating the oocyte, which is blocked at the metaphase of the second meiotic division (MII arrest) (6). It is known that deficiency in the PLC-zeta cascade leads to failure in oocyte activation and fertilization (7). Karabulut and colleagues have demonstrated a relationship between a sperm-associated oocyte-activating factor and PLC-zeta in the acrosome (8).

Artificial oocyte activation (AOA) is the most common strategy used for total fertilization failure and low fertilization (9). AOA is performed based on chemical, electrical, and mechanical protocols to overcome fertilization failure after ICSI (10). Ebner and colleagues suggested that AOA, using a ready-to-use compound, is a suitable means to overcome the problem of failed or impaired fertilization after ICSI (11). Also, Kochhar and colleagues reported successful pregnancy in two cases of total globozoospermia after ICSI with oocyte activation using a calcium ionophore (12). Other studies have revealed that AOA can improve the quality of embryos in ejaculated, epididymal, and testicular sperms (13, 14). There are benefits to using AOA solutions containing commercial ready-to-use ionophores (e.g., GM508 Cult-Active medium), including production under standard conditions with appropriate and effective concentrations of calcium, especially for immature sperm obtained from the testes tissue and epididymis (15). Ca ${}^{2+}$ ionophores in cult-active medium (CAM) transfer Ca ${}^{2+}$ and magnesium from the medium through the cell membrane into the oocyte and case to promote DNA synthesis and cell division (8).

In this prospective research, we evaluated a potentially positive influence of CAM to overcome oocyte activation deficiency and total fertilization failure in cases with severe male factor infertility such as OA.

## 2. Materials and Methods

### Participants

This case-control study was performed with 152 couples who were referred to the highly specialized infertility treatment center of the Academic Center for Education, Culture, and Research (ACECR) from August 2016 to March 2019. The couples suffered from severe male infertility (i.e., OA). The inclusion criteria were as follows: 1) female aged ≤ 35 yr; 2) no infertility in the female partner; 3) retrieval of at least five and at most 15 mature oocytes; and 4) injection of oocytes 36-40 hr after human chorionic gonadotropin (hCG) administration. Women with basal follicle-stimulating hormone (FSH) > 12 IU/mL, a history of ovarian surgery, or a history of endocrine disorders were excluded from the study.

The cases were divided into two groups: the control group, without exposure to CAM; and the CAM group, with the use of CAM after ICSI. Each group was further divided into two subgroups according to the origin of sperm retrieval for injection: 1) sperm were retrieved through testicular sperm extraction (TESE); and 2) sperm were retrieved through percutaneous epididymal sperm aspiration (PESA). 37 cycles were performed in the TESE-control subgroup, 38 in the PESA-control and PESA-CAM subgroups, and 39 in the TESE-CAM subgroup.

### ICSI performance

The ovulation induction protocol can be effectively applied for all cases as a standard long protocol. Accordingly, pituitary desensitization with a GnRH agonist (Buserelin, CinnaFact, Iran) and ovarian stimulation with gonadotropins (HMG, CinnaFact, Germany) were applied. A transvaginal ultrasound was performed for monitoring the cases. If at least three visible follicles measuring > 18 mm in diameter were observed on the ultrasound, HCG (10,000 IU, PDpreg, Germany) was injected. 36-40 hr post-triggering, oocytes were recovered and the standard ICSI protocol was performed.

All of the media used in this study were purchased from Origio, Denmark. Regardless of the sperm retrieval source, immediately after ICSI, injected oocytes in the CAM group were incubated for 15 min in 200 µL of GM 508 Cult-Active medium (Gynemed, Germany) (13). Following, treated oocytes were transferred to the fertilization medium.

### Fertilization, cleavage and embryo assessment

The fertilization rate was assessed 16-18 hr after ICSI by two distinct pronuclei (2PN) formation. Next, the embryos were transferred to the cleavage medium. The fertilization rate was defined as the proportion of total 2PN in mature oocytes in each group. The cleavage rate was determined as the ratio of total embryos / total 2PN in each group.

The embryo quality was graded on days two and three, based on the World Health Organization protocol and evaluated using an inverted microscope (OLYMPUS IX71, Tokyo, Japan). The following parameters were recorded in the analyses: 1) number of blastomeres; 2) percentage of fragmentation; 3) changes in blastomere symmetry; 4) presence of multinucleation; and 5) defects in zona pellucida and cytoplasm (16).

### Embryo transfer (ET) 

ET was performed under ultrasound guidance at 48-72 hr after oocyte retrieval. Depending on the embryo quality and woman's age, one-three embryos were transferred. ET was carried out transcervically using an embryo transfer catheter (Labotect, Gotting, Germany).

The primary outcomes included the number of mature oocytes, fertilization and cleavage rates, and quality of embryos, and the secondary outcomes were a chemical pregnancy, live birth and abortion. The fertilization rate was calculated based on the ratio of fertilized oocytes / the total number of injected MII oocytes.

Following pregnancy confirmation, administration of progesterone and estradiol valerate continued until the 10 ${}^{\text{th}}$ wk of pregnancy. Chemical pregnancy was defined as serum βhCG > 10 mIU/mL (VIDAS kit), measured 14 days after ET. Live birth was defined as any birth event in which at least one baby was born alive and survived for more than one month.

### Ethical considerations

This study was approved by the ACECR (Mashhad branch) Biomedical Ethics Committee (Code: IR.ACECR.JDM.REC.1398.002) and all couples signed a written consent form before the initiation of treatment cycles.

### Statistical analysis

Data were evaluated using the Statistical Package for the Social Sciences (SPSS) version 16. The variables were assessed in terms of normal distribution using the Kolmogorov-Smirnov test. Moreover, an independent sample *t* test was used for continuous normally distributed variables, while the Mann-Whitney U test was utilized for data that were not normally distributed. In addition, either Fisher's exact test or Chi-square test was used for the purpose of qualitative variables, as appropriate. P < 0.05 was regarded as statistically significant.

## 3. Results

### Participants

Using a prospective approach, oocytes were collected from 152 cases (152 cycles), resulting in 1643 cumulus-oocyte complexes. After denudation, only 84.29% (1385 out of 1643) of cumulus-oocyte complexes were mature. 857 oocytes in the CAM group and 786 oocytes in the control group were mature. In the control group, 50.6% and 49.1% of sperm were collected using PESA and TESE, respectively, while in the CAM group, the corresponding rates were 49.3% and 50.1%, respectively.

Statistically, all general characteristics of the CAM and control group were similar, regardless of whether sperm were retrieved from TESE or PESA (Table I). Consequently, they were considered equally appropriate for ICSI.

### Fertilization and cleavage rates 

To analyze the effect of CAM on fertilization rates, the oocytes were treated in the presence of CAM after ICSI. As indicated in table II, the fertilization rate in the PESA-CAM subgroup was higher compared with the PESA-control subgroup (55.00% ± 5.60 vs. 37.91% ± 4.88, p = 0.05); it was also higher in the TESE-CAM subgroup in comparison with the TESE-control subgroup (46.10% ± 4.41 vs. 36.50% ± 5.71, p = 0.02). The results showed that the cleavage rate under CAM treatment was significantly higher. More specifically, the cleavage rate was significantly higher in the PESA-CAM subgroup compared with the PESA-control subgroup (78.22% ± 3.42 vs. 55.31% ± 2.32, p ≤ 0.001) and was also higher in the TESE-CAM subgroup compared with the TESE-control subgroup (62.84% ± 3.20 vs. 50.10% ± 4.51, p = 0.04). The percentage of high-quality embryos in the PESA-CAM subgroup was significantly higher than in the PESA-control subgroup (66.23% ± 3.44 vs. 52.01% ± 3.14, p = 0.04).

However, no statistically significant difference was observed in the percentage of high-quality embryos between the TESE-CAM and TESE-control subgroups (55.10% ± 2.54 vs. 50.65 ± 2.11, p = 0.10).

### Embryo transfer and pregnancy rate

Overall, fewer cases in the CAM group had no embryo transfer (no-ET), in comparison with the control group. However, the percentage of no-ET in the PESA-CAM subgroup compared with the PESA-control subgroup did not differ significantly (p = 0.30), or in the TESE-CAM subgroup compared with the TESE-control subgroup (p = 0.34).

The rate of positive-βhCG pregnancy was slightly higher in the PESA-CAM subgroup than the control group (44% vs. 16%; p = 0.03). However, in the TESE-CAM subgroup, a significant difference was not observed compared with the TESE-control subgroup (29% vs. 13%; p = 0.24).

### Live birth and abortion

Live birth rates were significantly higher in the PESA-CAM subgroup compared with the PESA-control subgroup (35% vs. 10%; p = 0.01), but no significant difference was observed between the TESE-CAM and TESE-control subgroups (21% vs. 11%; p = 0.34). Also, the percentage of spontaneous abortions was lower in the CAM subgroups compared with their control subgroups. There was no significant difference in terms of gender. Finally, none of the babies born had major or minor congenital malformations. Only two cases of preterm delivery were reported.

**Table 1:** General characteristics related to the CAM and control groups


**Parameter**	**Control**		**CAM**	
	**PESA**	**TESE**	**PESA**	**TESE**
**Cycle number**	38	37	38	39
Female age (yr)*	29.23 ± 2.12	28.56 ± 2.22	28.65 ± 3.20	28.96 ± 2.32
Male age (yr)*	32.24 ± 3.50	33.45 ± 3.32	34.61 ± 2.45	35.12 ± 2.33
**Sperm retrieval (%)**	50.6	49.1	49.3	50.1
MII number/couple*	8.85 ± 5.06	8.12 ± 5.06	9.77 ± 5.31	8.65 ± 2.44
*Mean ± SD. PESA: Percutaneous epididymal sperm aspiration, TESE: Testicular sperm extraction, CAM: Cult-active medium				

**Table 2:** Comparison of ICSI outcomes between the CAM and control groups


**Groups**	**Number**	Fertilization rate (%)*	Cleavage rate (%)*	High-quality embryo (%)*	Pregnancy (%)**	No-ET (%)**	Abortion (%)**	Live birth (%)**
**PESA-CAM**	38	55.00 ± 5.60	78.22 ± 3.42	66.23 ± 3.44	44	8	18	35
**PESA-control**	38	37.91 ± 4.88	55.31 ± 2.32	52.01 ± 3.14	16	18	33	10
**p-value**	- 0.05	< 0.01	0.04	0.03	0.34	0.58	0.01
**TESE-CAM**	39	46.10 ± 4.41	62.84 ± 3.20	55.10 ± 2.54	29	10	27	21
**TESE-control**	37	36.50 ± 5.71	50.10 ± 4.51	50.65 ± 2.11	14	21	20	11
**p-value**	- 0.02	0.04	0.10	0.24	0.34	0.99	0.34
*Data are expressed as Mean ± SD. Independent *t* test. **Fisher's exact test. ET: Embryo transfer, PESA: Percutaneous epididymal sperm aspiration, TESE: Testicular sperm extraction, CAM: Cult-active medium								

## 4. Discussion

Although studies have shown that AOA can improve the outcomes of assisted reproductive technology (ART) in ICSI cases with severe male factor infertility due to sperm inability to activate the oocyte (1, 15), the use of an effective medium is still one of the most challenging research topics. Accordingly, the present study was performed to determine whether CAM as an AOA can improve ART results in OA infertile men.

Couples who are infertile due to OA suffer from fertilization failure in ICSI. Analyses of fertilization failure problems demonstrate that deficiency in the mechanism of oocyte activation is the most common cause. Recent studies have shown that defects in oocyte activation can be due to abnormalities in expression, localization or structure of PLC-zeta (17, 18).

Many researchers have tried different protocols, including chemical, electrical, and mechanical methods, to artificially activate oocytes to overcome fertilization failure after ICSI. Among these methods, chemical activation is the most commonly used method for AOA. No evidence of toxicity for this oocyte activation method has been reported (19). In addition, it is indicated that ICSI-AOA leads to no significant difference in the prevalence of major birth defects or types of birth defects (chromosomal aberrations and non-chromosomal aberrations) comparing with conventional ICSI (20). Consequently, the exposure of unfertilized oocytes to a combination of calcium ionophores and puromycin could effectively activate them within 20-68 hr after ICSI (19). Also, Kim and coworkers reported that pregnancy and childbirth using frozen-thawed embryos increased after calcium ionophore treatment (21). Mingrong and coworkers showed that AOA with a calcium ionophore (A23187) was able to improve the poor reproductive outcomes in certain types of infertile couples with a history of failure to achieve pregnancy (22).

Similarly to related studies, we reported that AOA with the use of CAM after ICSI resulted in significantly higher fertilization and cleavage rates. In addition, the results showed a difference in pregnancy and live birth rates in the PESA and TESE subgroups. PESA cases under CAM treatment had significantly better ICSI outcomes. There are two potential reasons for this: poor quality and insufficient maturity of sperm retrieved through TESE compared with PESA; and oocyte activation–sperm factor PLC-zeta may be at an insufficient concentration or may have an impaired function in immature spermatozoa retrieved through TESE compared with PESA.

Borges et al. reported that sperm retrieved from testes could not maintain calcium oscillations in the ooplasm after the artificial stimulation of oocyte activation (1). It has also been shown that an increase in intracytoplasmic calcium concentration during oocyte activation occurs in two steps (23, 24). The first step begins after gamete fusion, but the increase is not adequate for oocyte activation (25). In the second step, the sperm-associated oocyte-activating factor is released by sperm after fertilization and calcium oscillations occur, followed by oocyte activation (15, 26). Recently, a ready-to-use calcimycin solution (GM508 Cult-Active; Gynemed, Germany) was introduced for the clinical treatment of infertility, using an assisted reproductive method for azoospermia to overcome fertilization failure after ICSI (8, 15, 27). The present study showed that by fertilizing the oocytes after ICSI using CAM as an AOA medium, the fertilization rate could be significantly increased. Also, other related studies have reported a significant increase in the rate of fertilization, pregnancy, and live birth in cases under ICSI (8, 28, 29).

The results presented in table II showed that, not only was the fertilization rate higher in the groups treated with CAM, but also the cleavage rate. Thus, according to our findings, in cases of severe azoospermia, CAM can be used as an AO to improve fertilization and cleavage rates after ICSI. Our observations are in close agreement with Borges et al. (1) and Ebner et al. (15), who previously showed the positive effects of using CAM in improving ART outcomes. Also, other studies have reported an improvement in outcomes from the use of calcium ionophores as the main stimulant in CAM (30, 31). Our findings showed that even azoospermic cases could be successfully treated with CAM. Improvement of the fertilization rate and high-quality embryos in our study further led to significant improvements in the pregnancy rate.

In the present study, the rate of fertilization and pregnancy were higher in PESA cases receiving CAM compared to the group without exposure to the medium. Therefore, our results demonstrated the positive effects of GM508 Cult-Active medium on the enhancement of ICSI results in azoospermic cases by using epididymal and testicular spermatozoa for injection.

## 5. Conclusion

CAM could improve the fertilization and cleavage rates in OA cases. In particular, our study indicated that CAM had a significant effect on high embryo quality, and pregnancy and live birth rates in PESA cases. Routine use of GM508 Cult-Active medium as an AOA in ART centers requires further studies on the health of newborns.

##  Conflict of Interest 

The authors declare that they have no conflict of interest.
